# Poly[{μ_2_-1,2-bis­[4-(3-pyrid­yl)pyrimidin-2-ylsulfan­yl]ethane}di-μ_2_-cyanido-dicopper(I)]

**DOI:** 10.1107/S1600536808013172

**Published:** 2008-06-07

**Authors:** Ya-Wen Zhang, Hua-Ze Dong, Lin Cheng

**Affiliations:** a Department of Chemistry and Chemical Engineering, Southeast University, Nanjing, People’s Republic of China; b Department of Chemistry and Chemical Engineering, State Key Laboratory of Coordination Chemistry, Nanjing University, People’s Republic of China

## Abstract

The asymmetric unit of the title complex, [Cu_2_(CN)_2_(C_20_H_16_N_6_S_2_)]_*n*_, contains one Cu^I^ cation, one cyanide ligand and half of a centrosymmetric 1,2-bis­[4-(3-pyrid­yl)pyrimidin-2-ylsulfan­yl]ethane (bppe) ligand. The Cu^I^ atom displays a trigonal coordination geometry, being surrounded by one C atom from one cyanide anion and two N atoms from one cyanide and one bppe ligand. In the complex, each cyanide anion links two Cu^I^ atoms in a bis-monodentate mode into a zigzag [–Cu—CN–]_*n*_ chain. Two parallel chains are linked by bppe ligands into a ladder chain.

## Related literature

For related literature, see: Awaleh *et al.* (2005 ▶); Bu *et al.* (2003 ▶); Chen *et al.* (2003 ▶); Su *et al.* (2000 ▶); Xie *et al.* (2005 ▶).
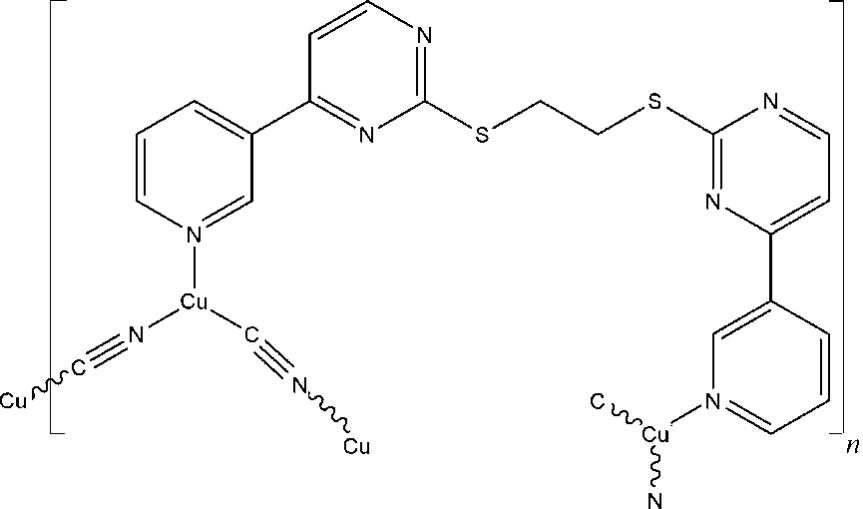


## Experimental

### 

#### Crystal data

[Cu_2_(CN)_2_(C_20_H_16_N_6_S_2_)]
*M* *_r_* = 291.81
Monoclinic, 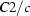

*a* = 16.025 (4) Å
*b* = 16.296 (7) Å
*c* = 9.3103 (17) Å
β = 105.660 (19)°
*V* = 2341.1 (12) Å^3^
*Z* = 8
Mo *K*α radiation
μ = 2.02 mm^−1^
*T* = 153 (2) K
0.50 × 0.20 × 0.10 mm

#### Data collection

Bruker APEX CCD diffractometer
Absorption correction: multi-scan (*SADABS*; Sheldrick, 2002 ▶) *T*_min_ = 0.431, *T*_max_ = 0.823
6130 measured reflections
2290 independent reflections
1925 reflections with *I* > 2σ(*I*)
*R*_int_ = 0.036

#### Refinement

*R*[*F* ^2^ > 2σ(*F* ^2^)] = 0.032
*wR*(*F* ^2^) = 0.083
*S* = 1.09
2290 reflections
154 parameters
H-atom parameters constrained
Δρ_max_ = 0.41 e Å^−3^
Δρ_min_ = −0.32 e Å^−3^

### 

Data collection: *SMART* (Bruker, 2000 ▶); cell refinement: *SMART*; data reduction: *SAINT-Plus* (Bruker, 2000 ▶); program(s) used to solve structure: *SHELXTL* (Sheldrick, 2008 ▶); program(s) used to refine structure: *SHELXTL*; molecular graphics: *SHELXTL*; software used to prepare material for publication: *SHELXTL*.

## Supplementary Material

Crystal structure: contains datablocks I, global. DOI: 10.1107/S1600536808013172/hg2393sup1.cif

Structure factors: contains datablocks I. DOI: 10.1107/S1600536808013172/hg2393Isup2.hkl

Additional supplementary materials:  crystallographic information; 3D view; checkCIF report

## Figures and Tables

**Fig. 1.**
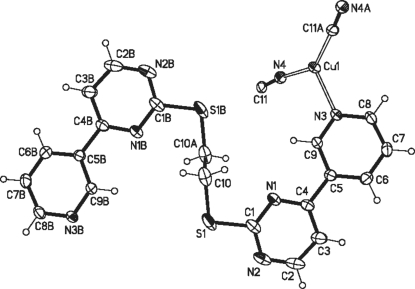
Local coordination environment of the title compound with 30% thermal ellipsoids. All the hydrogen atoms are omitted for clarity. Symmetry codes for 1, a: x, -y, 1/2 + z; b: 1 - x, -y,1 - z.

**Fig. 2.**
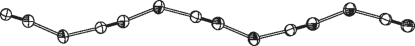
The zigzag (CuCN)n chain in the title compound.

**Fig. 3.**
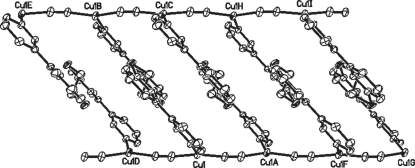
The one-dimensional ladder chain of the title compound.

## supplementary crystallographic information

### Comment

There has been current significant interest in the rational design and synthesis of metal-organic coordination architectures by using flexible bridging units due that the flexibility and conformational freedoms of such ligands offer the possibility for the construction of unprecedented frameworks (Su *et al.*, 2000). Recently, flexible thioethers have been well established ligands in coordination and metallosupramolecular chemistry because of their rich structural information (Awaleh *et al.*,  2005, Bu *et al.*,  2003, Chen *et al.*,  2003, Xie *et al.*,  2005). Herein, we report the crystal structure of the title compound, [Cu_2_(CN)_2_(C_20_H_16_N_6_S_2_)]_n_, based on a pyridyl dithioether ligand–1,2-bis(4-(pyridinyl-4-)pyrimidin-2-ylthio)ethane. The asymmetric unit of the title complex, contains one Cu^I^ cation, one cyano and half a bppe (bppe = 1,2-bis(4-(pyridinyl-4-)pyrimidin -2-ylthio)ethane) ligand. The Cu^I^ atom displays a triangular geometry, being surrounded by one carbon atom (Cu1—C11*a* 1.873 (3) Å) from one cyano anion and two nitrogen atoms from one cyano (Cu1—N4 1.916 (2) Å) and one bppe ligand (Cu1—N3 1.873 (3) Å). In the complex, each cyano aion links two Cu^I^ atoms in a bis-monodentate mode into a zigzag (CuCN)_n_ chain. The shortest intrachain Cu—Cu distance is 4.894 (2) Å. Two parallel zigzag chains were linked by bppe ligands into a one-dimensional ladder chain, in which the Cu—Cu distance separated by bppe is 11.648 (3) Å. The ladder chain is stabilized by the intraladder C–H···N hydrogen bonds (C9—N1 2.810 (3) Å; C8—N2 3.398 (4) Å). Finally, the ladder chains were constructed into a three-dimensional supramolecular network by the interladder C10–H···N1c (c = -1/2 + *x*,1/2 - *y*,1/2 + *z*) hydrogen bond with the C···N distance 2.891 (4) Å.

### Experimental

A mixture of bppe (0.040 g, 0.1 mmol), CuCN (0.018 g, 0.2 mmol), and water (6 ml) were heated in a 15-ml Teflon-lined vessel at 403 K for 3 days, followed by slow cooling (5 K/hr) to room temperature. After filtration and washing with H2O, colorless needle-like crystals were collected and dried in air (0.019 g, yield *ca* 32% based on bppe).

### Refinement

H atoms were positioned geometrically and refined using a riding model, with C—H = 0.93–0.97 Å and with *U*_iso_(H) = 1.2.

### Crystal data

**Table:**

[Cu_2_(CN)_2_(C_20_H_16_N_6_S_2_)]	*F*_000_ = 1176
*M**_r_* = 291.81	*D*_x_ = 1.656 Mg m^−^^3^
Monoclinic, *C*2/*c*	Mo *K*α radiation λ = 0.71073 Å
Hall symbol: -C 2yc	Cell parameters from 765 reflections
*a* = 16.025 (4) Å	θ = 2.5–28.0º
*b* = 16.296 (7) Å	µ = 2.02 mm^−^^1^
*c* = 9.3103 (17) Å	*T* = 153 (2) K
β = 105.660 (19)º	Needle-like, colorless
*V* = 2341.1 (12) Å^3^	0.50 × 0.20 × 0.10 mm
*Z* = 8	

### Data collection

**Table:**

Bruker APEX CCD diffractometer	2290 independent reflections
Radiation source: fine-focus sealed tube	1925 reflections with *I* > 2σ(*I*)
Monochromator: graphite	*R*_int_ = 0.036
*T* = 153(2) K	θ_max_ = 26.0º
φ and ω scans	θ_min_ = 2.5º
Absorption correction: multi-scan(SADABS; Sheldrick, 2002)	*h* = −19→9
*T*_min_ = 0.431, *T*_max_ = 0.823	*k* = −20→19
6130 measured reflections	*l* = −11→11

### Refinement

**Table:**

Refinement on *F*^2^	Secondary atom site location: difference Fourier map
Least-squares matrix: full	Hydrogen site location: inferred from neighbouring sites
*R*[*F*^2^ > 2σ(*F*^2^)] = 0.032	H-atom parameters constrained
*wR*(*F*^2^) = 0.083	*w* = 1/[σ^2^(*F*_o_^2^) + (0.0456*P*)^2^ + 0.02*P*] where *P* = (*F*_o_^2^ + 2*F*_c_^2^)/3
*S* = 1.09	(Δ/σ)_max_ < 0.001
2290 reflections	Δρ_max_ = 0.41 e Å^−^^3^
154 parameters	Δρ_min_ = −0.32 e Å^−^^3^
Primary atom site location: structure-invariant direct methods	Extinction correction: none

### Special details

**Table:**

Geometry. All e.s.d.'s (except the e.s.d. in the dihedral angle between two l.s. planes) are estimated using the full covariance matrix. The cell e.s.d.'s are taken into account individually in the estimation of e.s.d.'s in distances, angles and torsion angles; correlations between e.s.d.'s in cell parameters are only used when they are defined by crystal symmetry. An approximate (isotropic) treatment of cell e.s.d.'s is used for estimating e.s.d.'s involving l.s. planes.
Refinement. Refinement of *F*^2^ against ALL reflections. The weighted *R*-factor *wR* and goodness of fit *S* are based on *F*^2^, conventional *R*-factors *R* are based on *F*, with *F* set to zero for negative *F*^2^. The threshold expression of *F*^2^ > σ(*F*^2^) is used only for calculating *R*-factors(gt) *etc*. and is not relevant to the choice of reflections for refinement. *R*-factors based on *F*^2^ are statistically about twice as large as those based on *F*, and *R*- factors based on ALL data will be even larger.

### Fractional atomic coordinates and isotropic or equivalent isotropic displacement parameters (Å^2^)

**Table:**

	*x*	*y*	*z*	*U*_iso_*/*U*_eq_	
Cu1	0.200534 (18)	0.046307 (17)	0.73287 (3)	0.04397 (13)	
S1	0.58681 (4)	0.09504 (5)	0.45165 (9)	0.0681 (2)	
N1	0.46673 (11)	0.17752 (12)	0.5455 (2)	0.0424 (4)	
N2	0.55869 (14)	0.25077 (17)	0.4267 (2)	0.0624 (6)	
N3	0.25417 (11)	0.16006 (11)	0.71533 (19)	0.0379 (4)	
N4	0.18704 (14)	0.00114 (12)	0.5379 (2)	0.0506 (5)	
C1	0.52978 (14)	0.18272 (17)	0.4778 (3)	0.0496 (6)	
C2	0.52069 (18)	0.3195 (2)	0.4501 (3)	0.0661 (8)	
H2	0.5387	0.3684	0.4167	0.079*	
C3	0.45586 (16)	0.32250 (16)	0.5214 (3)	0.0539 (6)	
H3	0.4308	0.3719	0.5373	0.065*	
C4	0.42959 (13)	0.24839 (14)	0.5686 (2)	0.0386 (5)	
C5	0.35930 (13)	0.24308 (13)	0.6430 (2)	0.0355 (5)	
C6	0.33133 (15)	0.31071 (14)	0.7073 (3)	0.0452 (5)	
H6	0.3570	0.3617	0.7045	0.054*	
C7	0.26579 (16)	0.30235 (15)	0.7749 (3)	0.0514 (6)	
H7	0.2461	0.3475	0.8174	0.062*	
C8	0.22945 (14)	0.22636 (15)	0.7791 (3)	0.0442 (5)	
H8	0.1862	0.2206	0.8279	0.053*	
C9	0.31811 (13)	0.16915 (13)	0.6502 (2)	0.0367 (5)	
H9	0.3361	0.1233	0.6071	0.044*	
C10	0.54252 (17)	0.01639 (19)	0.5480 (3)	0.0632 (7)	
H10A	0.5837	−0.0283	0.5753	0.076*	
H10B	0.5337	0.0392	0.6390	0.076*	
C11	0.18755 (15)	−0.01827 (14)	0.4207 (3)	0.0432 (5)	

### Atomic displacement parameters (Å^2^)

**Table:**

	*U*^11^	*U*^22^	*U*^33^	*U*^12^	*U*^13^	*U*^23^
Cu1	0.0547 (2)	0.0449 (2)	0.04047 (19)	−0.00041 (12)	0.02691 (14)	0.00271 (11)
S1	0.0465 (4)	0.0949 (6)	0.0744 (5)	0.0002 (4)	0.0360 (3)	−0.0228 (4)
N1	0.0357 (10)	0.0536 (12)	0.0422 (10)	−0.0013 (8)	0.0179 (8)	−0.0022 (9)
N2	0.0467 (13)	0.0923 (19)	0.0555 (14)	−0.0152 (12)	0.0262 (11)	0.0025 (12)
N3	0.0387 (10)	0.0410 (10)	0.0402 (10)	−0.0013 (8)	0.0212 (8)	−0.0016 (8)
N4	0.0740 (14)	0.0401 (11)	0.0468 (11)	0.0020 (10)	0.0320 (10)	0.0010 (9)
C1	0.0354 (13)	0.0772 (18)	0.0396 (12)	−0.0048 (11)	0.0163 (10)	−0.0068 (12)
C2	0.0510 (16)	0.082 (2)	0.0691 (18)	−0.0173 (15)	0.0228 (14)	0.0213 (16)
C3	0.0458 (14)	0.0561 (15)	0.0623 (16)	−0.0030 (11)	0.0190 (12)	0.0152 (12)
C4	0.0291 (11)	0.0495 (13)	0.0379 (11)	−0.0011 (9)	0.0101 (9)	0.0048 (9)
C5	0.0325 (11)	0.0379 (12)	0.0370 (11)	0.0017 (8)	0.0113 (9)	0.0054 (8)
C6	0.0444 (13)	0.0359 (12)	0.0571 (14)	−0.0006 (9)	0.0167 (11)	0.0006 (10)
C7	0.0514 (15)	0.0442 (14)	0.0643 (16)	0.0045 (11)	0.0253 (12)	−0.0131 (12)
C8	0.0409 (13)	0.0515 (14)	0.0474 (13)	0.0023 (10)	0.0241 (10)	−0.0046 (11)
C9	0.0390 (12)	0.0361 (11)	0.0401 (11)	0.0028 (9)	0.0193 (9)	0.0003 (9)
C10	0.0495 (15)	0.0784 (19)	0.0614 (16)	0.0148 (14)	0.0144 (12)	−0.0181 (15)
C11	0.0614 (15)	0.0351 (11)	0.0401 (12)	0.0082 (10)	0.0259 (11)	0.0038 (10)

### Geometric parameters (Å, °)

**Table:**

Cu1—C11^i^	1.873 (2)	C3—H3	0.9300
Cu1—N4	1.916 (2)	C4—C5	1.476 (3)
Cu1—N3	2.0683 (19)	C5—C9	1.384 (3)
S1—C1	1.748 (3)	C5—C6	1.385 (3)
S1—C10	1.815 (3)	C6—C7	1.369 (4)
N1—C1	1.330 (3)	C6—H6	0.9300
N1—C4	1.343 (3)	C7—C8	1.374 (3)
N2—C2	1.321 (4)	C7—H7	0.9300
N2—C1	1.338 (3)	C8—H8	0.9300
N3—C9	1.332 (3)	C9—H9	0.9300
N3—C8	1.343 (3)	C10—C10^ii^	1.511 (5)
N4—C11	1.138 (3)	C10—H10A	0.9700
C2—C3	1.376 (4)	C10—H10B	0.9700
C2—H2	0.9300	C11—Cu1^iii^	1.873 (2)
C3—C4	1.389 (3)		
			
C11^i^—Cu1—N4	141.12 (9)	C9—C5—C6	117.2 (2)
C11^i^—Cu1—N3	116.47 (8)	C9—C5—C4	120.5 (2)
N4—Cu1—N3	102.27 (8)	C6—C5—C4	122.2 (2)
C1—S1—C10	102.68 (12)	C7—C6—C5	119.8 (2)
C1—N1—C4	116.6 (2)	C7—C6—H6	120.1
C2—N2—C1	115.1 (2)	C5—C6—H6	120.1
C9—N3—C8	117.83 (19)	C6—C7—C8	119.1 (2)
C9—N3—Cu1	121.50 (14)	C6—C7—H7	120.4
C8—N3—Cu1	120.50 (15)	C8—C7—H7	120.4
C11—N4—Cu1	170.7 (2)	N3—C8—C7	122.3 (2)
N1—C1—N2	127.0 (2)	N3—C8—H8	118.8
N1—C1—S1	120.4 (2)	C7—C8—H8	118.8
N2—C1—S1	112.58 (18)	N3—C9—C5	123.65 (19)
N2—C2—C3	123.5 (3)	N3—C9—H9	118.2
N2—C2—H2	118.3	C5—C9—H9	118.2
C3—C2—H2	118.3	C10^ii^—C10—S1	111.6 (3)
C2—C3—C4	117.0 (3)	C10^ii^—C10—H10A	109.3
C2—C3—H3	121.5	S1—C10—H10A	109.3
C4—C3—H3	121.5	C10^ii^—C10—H10B	109.3
N1—C4—C3	120.8 (2)	S1—C10—H10B	109.3
N1—C4—C5	116.88 (19)	H10A—C10—H10B	108.0
C3—C4—C5	122.3 (2)	N4—C11—Cu1^iii^	173.9 (2)

Symmetry codes: (i) *x*, −*y*, *z*+1/2; (ii) −*x*+1, −*y*, −*z*+1; (iii) *x*, −*y*, *z*−1/2.
